# Succinate Mediates Tumorigenic Effects *via* Succinate Receptor 1: Potential for New Targeted Treatment Strategies in Succinate Dehydrogenase Deficient Paragangliomas

**DOI:** 10.3389/fendo.2021.589451

**Published:** 2021-03-12

**Authors:** Dieter M. Matlac, Katerina Hadrava Vanova, Nicole Bechmann, Susan Richter, Julica Folberth, Hans K. Ghayee, Guang-Bo Ge, Luma Abunimer, Robert Wesley, Redouane Aherrahrou, Margo Dona, Ángel M. Martínez-Montes, Bruna Calsina, Maria J. Merino, Markus Schwaninger, Peter M. T. Deen, Zhengping Zhuang, Jiri Neuzil, Karel Pacak, Hendrik Lehnert, Stephanie M. J. Fliedner

**Affiliations:** ^1^Neuroendocrine Oncology and Metabolism, Medical Department I, Center of Brain, Behavior, and Metabolism, University Medical Center Schleswig-Holstein Lübeck, Lübeck, Germany; ^2^Institute of Biotechnology, Czech Academy of Sciences, Prague-West, Czechia; ^3^Section on Medical Neuroendocrinology, Eunice Kennedy Shriver National Institute of Child Health and Human Development, National Institutes of Health, Bethesda, MD, United States; ^4^Institute of Clinical Chemistry and Laboratory Medicine, University Hospital Carl Gustav Carus, Medical Faculty Carl Gustav Carus, Technische Universität Dresden, Dresden, Germany; ^5^Institute for Experimental and Clinical Pharmacology and Toxicology, University of Lübeck, Lübeck, Germany; ^6^Department of Medicine, Division of Endocrinology, University of Florida and Malcom Randall VA Medical Center, Gainesville, FL, United States; ^7^Institute of Interdisciplinary Integrative Medicine Research, Shanghai University of Traditional Chinese Medicine, Shanghai, China; ^8^Institute for Cardiogenetics, University of Lübeck, Lübeck, Germany; ^9^Department of Biomedical Engineering, Centre for Public Health Genomics, University of Virginia, Charlottesville, VA, United States; ^10^Division of Endocrinology 471, Department of Internal Medicine, Radboud University Medical Center, Nijmegen, Netherlands; ^11^Hereditary Endocrine Cancer Group, Human Cancer Genetics Program, Spanish National Cancer Research Centre (CNIO), Madrid, Spain; ^12^Laboratory of Surgical Pathology, National Cancer Institute, National Institutes of Health, Bethesda, MD, United States; ^13^Radboud University, Nijmegen, Netherlands; ^14^Surgical Neurology Branch, National Institute of Neurological Disorders and Stroke, National Institutes of Health, Bethesda, MD, United States; ^15^School of Medical Science, Griffith University, Southport, QLD, Australia

**Keywords:** succinate receptor 1, SUCNR1 (GPR91), paraganglioma, succinate, SDHB gene

## Abstract

Paragangliomas and pheochromocytomas (PPGLs) are chromaffin tumors associated with severe catecholamine-induced morbidities. Surgical removal is often curative. However, complete resection may not be an option for patients with succinate dehydrogenase subunit A-D (*SDHx*) mutations. *SDHx* mutations are associated with a high risk for multiple recurrent, and metastatic PPGLs. Treatment options in these cases are limited and prognosis is dismal once metastases are present. Identification of new therapeutic targets and candidate drugs is thus urgently needed. Previously, we showed elevated expression of succinate receptor 1 (*SUCNR1*) in *SDHB* PPGLs and *SDHD* head and neck paragangliomas. Its ligand succinate has been reported to accumulate due to *SDHx* mutations. We thus hypothesize that autocrine stimulation of SUCNR1 plays a role in the pathogenesis of *SDHx* mutation-derived PPGLs. We confirmed elevated SUCNR1 expression in *SDHx* PPGLs and after *SDHB* knockout in progenitor cells derived from a human pheochromocytoma (hPheo1). Succinate significantly increased viability of *SUCNR1*-transfected PC12 and ERK pathway signaling compared to control cells. Candidate *SUCNR1* inhibitors successfully reversed proliferative effects of succinate. Our data reveal an unrecognized oncometabolic function of succinate in *SDHx* PPGLs, providing a growth advantage *via SUCNR1*.

## Introduction

Paragangliomas (PGLs) are catecholamine-producing chromaffin tumors of the autonomic nervous system, including adrenal-derived pheochromocytomas (together PPGLs). While curative in the majority of cases, resection is not an option for many paragangliomas with loss-of-function mutations of succinate dehydrogenase (*SDH*) subunits *A-D* (summarized as *SDHx*). Particularly mutations in the *SDHB* gene predispose to metastases (34–69%) (1–4), usually making complete resection impossible. Mutations in *SDHA*, *SDHC*, and *SDHD* subunits predominantly cause head and neck PGLs (HNPs) (5–7), which can be inoperable due to proximity to vital structures such as vessels or nerves. In addition, surgical complication rate is high, particularly for carotid body location, causing nerve damage in 48% of cases, including 17% with permanent damage (8). Also for *SDHA*, *SDHC*, and *SDHD* mutations, metastatic disease has been reported (9). Treatment options for inoperable cases are extremely limited and prognosis is dismal once metastases are present. Thus, identification of new therapeutic targets and candidate drugs is urgently needed.

*SDHx*-PGLs are characterized by dysfunction of the SDH enzyme. The conversion of succinate to fumarate is impaired, causing substantial succinate accumulation (10–13). Similarly, reduced SDH activity and succinate accumulation has been associated with progressive disease or poor outcome in endometrial cancer (14) and hepatocellular carcinoma (15). Accumulated succinate can cross both the inner and outer mitochondrial membrane *via* the dicarboxylic acid transporter and the voltage-dependent anion channel (VDAC) [summarized in (12, 16)] to reach the cytosol. There, excess succinate mediates oncogenic effects by inhibition of 2-oxoglutarate-dependent prolyl hydroxylases and demethylases (17). Obstruction of prolyl hydroxylation of hypoxia inducible transcription factors (HIFs) prevents their degradation and induces expression of tumor promoting HIF-target genes. Moreover, inhibition of DNA and histone demethylases causes hypermethylation, which represses transcription of affected genes. Despite knowledge of the underlying mechanisms, targeted treatment approaches for mostly inoperable *SDHx*-PPGL are still lacking.

In addition to its established role as an oncometabolite, succinate has also been recognized to act as a ligand for the G-protein-coupled receptor succinate receptor 1 (SUCNR1/GPR91) (18). Elevations in succinate levels arise during hypoxia/ischemia, hyperglycemia, due to tissue damage, or at sites of inflammation [summarized in (19)]. More recently, pH dependent transport of succinate from intact cells *via* monocarboxylate transporter 1 has been shown in an ischemia reperfusion model of the heart and following exercise under acidic conditions (20, 21). An apparent function of SUCNR1 is the activation of coping mechanisms upon adverse conditions, including stimulation of proliferation of different cell types, migration, and angiogenesis (22–29).

Cancer promoting effects of succinate-SUCNR1 signaling have recently been recognized, and include induction of epithelial to mesenchymal transition, migration, and metastatic spread of lung cancer cells as well as immunosuppressive effects (30). Involvement of SUCNR1 in tumor angiogenesis has also been proposed (31).

Depending on cell type, the effects of SUCNR1 stimulation are conveyed by different mechanisms, at least in part related to G-protein coupling. In kidney cells, coupling to Gαq- and/or Gαi-proteins has been proposed, leading to activation of extracellular-signal-regulated-kinases (ERK), generation of inositol triphosphate, augmentation of intracellular calcium, and decrease of cyclic adenosine monophosphate (cAMP) (25). Some authors suggested that calcium mobilization is rather mediated by the βγ dimers than coupling to Gαq (26). In cardiomyocytes, SUCNR1 stimulation has been shown to increase cAMP concentration, thus coupling to Gαs is also possible (25).

Among a range of different tissues (32) *Sucnr1* has also been observed in the mouse adrenal (33) and chromaffin cells of the carotid body (34). Its role in chromaffin cells and chromaffin cell-derived PPGLs however is not yet clear.

Succinate treatment as well as *SDHB*-silencing has been shown to induce *SUCNR1* mRNA and protein expression in human hepatoma cells (35), suggesting a positive feedback of inappropriate succinate accumulation on expression of its receptor. Consistently, we detected elevated *SUCNR1* expression in *SDHB* PPGLs and *SDHD* HNPs (36). We thus hypothesized that a combination of abundant succinate and its receptor SUCNR1 is a unique characteristic of *SDHx*-mutated tumors, which highly likely contributes to tumor formation, growth, or spread. Potent and selective small molecule inhibitors for SUCNR1 have been previously described (37). Targeting SUCNR1 thus represents a promising new therapeutic strategy for *SDHx* PPGLs.

## Material and Methods

### Human Tissue

Fresh PPGL tissue was collected at the National Institutes of Health in Bethesda, MD, USA, under a protocol approved by the *Eunice Kennedy Shriver* National Institute of Child Health and Human Development’s Institutional Review Board. Previous to tissue collection, patients gave informed written consent in accordance with the protocol. Tumor tissue was partially fixed in 4% formalin for subsequent paraffin embedding.

### Immunohistochemistry

Paraffin was removed from the tissues after warming slides to 60°C with xylene. Tissue was rehydrated stepwise in decreasing ethanol concentrations and epitopes were retrieved in heated citrate buffer (10 mM sodium citrate, 1 mM citric acid, pH 6). Tris-buffered saline with 0.1% tween 20 was used for wash steps. Endogenous peroxidases were inhibited with 3% H_2_O_2_ followed by DAKO protein block serum-free (Dako, Glostrup, Denmark). Slides were incubated with rabbit anti-SUCNR1 antibody (ab140795 Abcam, Cambridge, UK) in blocking solution in a humidified chamber for 1 h at 37°C. Peroxidase-labeled polymer conjugated with secondary goat anti-rabbit antibody (Dako EnVision) was applied. Visualization was based on the peroxidase reaction with 3,3-diaminobenzidine solution (Dako). Tissue was counterstained with hematoxylin. Dehydration was performed by stepwise immersion in increasing ethanol concentrations followed by xylene before mounting.

### SUCNR1 Expression Analysis

mRNA data from 227 tumors was extracted from gene expression array (38–40) and RNAseq datasets (41) using a data analysis pipeline as detailed elsewhere (42). One-tailed Mann-Whitney test was applied to test for differences in *SUCNR1* expression between *SDHx* and cluster 2 PPGLs (*RET*, *MAX*, *NF1*, *TMEM127*, *FGFR1*, and *HRAS*) in the different series.

### Cell Culture

Rat pheochromocytoma cells (PC12) and mouse tumor tissue cells silenced for *Sdhb* (MTTCtr, MTTsh*Sdhb*63, MTTsh*Sdhb*64) (43) were cultured at 37°C with 5% CO_2_ in DMEM with 4.5 g/L glucose, 4.5 g/L L-glutamine without pyruvate (Gibco, Grand Island, NY, USA) supplemented with 10% heat-inactivated horse serum (Biowest, Nuaillé, France), 5% fetal bovine serum (BioWhittaker, Lonza, Basel, Switzerland). For PC12 1% penicillin/streptomycin (Merck, Darmsadt, Germany) was added to the media, while MTTCtr, MTTsh*Sdhb*63, MTTsh*Sdhb*64 were grown in presence of 1 µg/ml puromycin (InvivoGen Euorpe, Toulouse, France) to suppress untransfected cells. Oxygen deprivation experiments and collection of cells were performed in an InvivO_2_ workstation (Baker, Sanford, ME, USA) at the indicated oxygen concentrations.

### hPheo1 *SDHB* Knockout

Progenitor cells derived from a human pheochromocytoma (hPheo1) were used. Genomic deletion of *SDHB* in hPheo1 cells was performed by the CRISPR/AsCPF1 system (44) using the pX AsCpf1-Venus-NLS crRNA entry plasmid. Suitable guide RNAs were identified using the Crispor software. An oligo was designed containing an overhang for plasmid insertion, followed by an array of three guide RNAs targeting before (TATCCAGCGTTACATCTGTTGTG), inside (CCATCTATCGATGGGACCCAGAC), and after (GCTTTTCACATCCTTGGAAGGCT) exon 2 of human SDHB, separated by the AsCpf1 direct repeat sequences: AGATTATCCAGCGTTACATCTGTTGTGAATTTCTACTCTTGTAGATCCATCTATCGATGGGACCCAGACAATTTCTACTCTTGTAGATGCTTTTCACATCCTTGGAAGGCT. The oligo was cloned into the plasmid cleaved by FastDigest BpiI (Thermo Fisher) and the correct insertion was confirmed by colony PCR and DNA sequencing. hPheo1 cells were transfected with the verified CPF1 construct using Lipfectamine3000 (Thermo Fisher), followed by single cell sorting for Venus-positive cells into a 96-well culture plate. Clones were collected and deletion of the targeted locus was confirmed by genomic PCR using primers ACTTTCCCAACAGTATCGCTCTT and TCAAGGCAAGTTTCTGGCGGT. SDHB knockout clones were confirmed by western blotting for SDHB and DNA sequencing. Human SDHB was re-expressed in *SDHB* KO cells from the pLYS5-SDHB-Flag plasmid (Addgene # 50055, a kind gift of Vamsi Mootha) using lentiviral transduction. Lentivirus particles were produced in Hek293T cells using second generation psPAX and pMD.2G plasmids and Lipofectamine3000. Virus-containing media were collected after 48 h, centrifuged at 3,000 × g for 15 min and stored at −80°C.

hPheo1 parental cells (Ctr) and *SDHB* KO (*SDHB*^KO23^) or re-expressing cells (*SDHB*^KO23Rec^) were kept in RPMI (Life technologies, Darmstadt, Germany) with 10% FBS (BioWhittaker), 1% penicillin/streptomycin (Merck, Darmsadt, Germany), 4.5 g/L glucose, 2mM sodium pyruvate, and 50 µg/ml uridine (Sigma-Aldrich, Saint Louis, MO, USA). *SDHB*^KO23Rec^ received 50 µg/ml hygromycin B (Th. Geyer, Hamburg, Germany).

### Evaluation of Oxygen Consumption Rate

The Seahorse XF96 Extracellular Flux Analyzer was used for assessment of cellular oxygen consumption rate (OCR) following the manufacturer’s instructions. Briefly, all hPheo1 cells were seeded in poly-L-lysine coated XF96 cell culture microplates at 5 × 10^3^ per well in standard culture media. After 24 h, the medium was replaced by serum-free DMEM containing 10 mM glucose, 2mM L-glutamine, 1 mM pyruvate, and 5 mM HEPES, pH 7.4. After equilibration of temperature and pH for 30 min at 37°C mitochondrial respiration was determined in consecutive injection steps [1 μM oligomycin (OMY), 1.5 μM CCCP, and a combination of 0.5 μM rotenone (ROT) and 0.5 μM antimycin A (AMA)]. OCR measurements were made using the manufacturer’s setting. As last injection, Hoechst 33432 was added (2 μg/ml) and the number of cells was evaluated by MD ImageXpress Micro XLS. Results were analyzed by the XF Stress Test Report Generators (Agilent Technologies) and normalized to cell count.

### Mass Spectrometric Analysis of Krebs Cycle Metabolites

hPheo1-Ctr, -*SDHB*^KO23^ and -*SDHB*^KO23Rec^ (300,000 cells/well) or MTTCtr, MTTsh*Sdhb*63, MTTsh*Sdhb*64 (500,000 cells/well) were seeded into rat tail collagen-coated six-well plates. MTTCtr, MTTsh*Sdhb*63, MTTsh*Sdhb*64 were grown under hypoxic conditions (1 and 10% O_2_) and cells from the same passage were kept at normoxia (N1 and N10). Cells were harvested in ice-cold methanol. Extracts were centrifuged, dried down using a SpeedVac concentrator (Thermo Scientific) and MTTCtr, MTTsh*Sdhb*63, MTTsh*Sdhb*64 metabolites were resuspended in mobile phase for subsequent quantification by ultra high-pressure liquid chromatography tandem mass spectrometry (LC-MS/MS) as described previously (11).

Conditioned media from hPheo1-Ctr, -*SDHB*^KO23^ and -*SDHB*^KO23Rec^ were collected previous to cell lysis in methanol. Extracts and media were dried down using a SpeedVac concentrator (Thermo Scientific) and metabolites were re-suspended in methanol at 10-fold concentration, agitated at 600 rpm and 4°C for 10 min, followed by centrifugation at 20,000 × g for 10 min at 4°C. Relative quantification of metabolites in the supernatant was performed on a LC-MS/MS system, consisting of a Dionex Ultimate 3000 RS LC-system coupled to an Orbitrap mass spectrometer (QExactive, ThermoFisher Scientific, Bremen, Germany) equipped with a heated-electrospray ionization (HESI-II) probe. A Waters Acquity UPLC BEH Amide column (2.1 × 100 mm, 2.5 µm), maintained at 40°C, was used for chromatographic separation. Mobile phases consisted of (A) 0.1% formic acid in water and (B) 0.1% formic acid in acetonitrile with a flow rate of 0.2 ml/min. Following gradient was applied: 75% B to 70% B in 0.5 min and to 65% B in 1.0 min. Final step to 60% B in another 0.5 min, held for 1.0 min and back to 75% B in 0.1 min. Equilibration time was 1.9 min. A parallel reaction monitoring (PRM) experiment in the negative ionization mode was used for the targeted analysis of succinate and fumarate. Mass resolution was 70,000, the isolation window was set to 1.5 m/z. PRM transitions and scan parameters are shown in **Table S1**.

### PC12 Cell Transfection

PC12 cells were seeded into collagen-1-coated 96-well plates (Corning Biocoat, Kaiserslautern, Germany). Lipofectamine3000 was used to transfect PC12 cells with a pmCherry-N1 vector encoding a fusion protein of mCherry and human *SUCNR1* or enhanced green fluorescent protein (*EGFP*) following manufacturer recommendations. Plasmids were generously provided by Prof. Deen. Geneticin resistance allowed selection of stable clones in presence of 1 mg/ml geneticin (Roth, Karlsruhe, Germany). Since propagation of PC12 from single clones was not possible, multiclonal cultures were used.

### Quantitative Real-Time Polymerase Chain Reaction

Cells were collected in NucleoSpin RNA mini kit lysis buffer and RNA extraction was performed according to the manufacturer’s manual (Macherey-Nagel, Düren, Germany). For cDNA synthesis the SuperScript™ III First-Strand Synthesis SuperMix has been used (Thermo Fisher). Quantitative RT-PCR was performed on a Quant studio 5 instrument (Thermo Fisher) using SYBR green PCR Master mix (Thermo Fisher), following the recommended cycling conditions. Used primers are listed in **Table S2**.

### Western Blot

Stably SUCNR1- and EGFP-expressing cells were seeded into 10 cm collagen-1-coated cell culture dishes at 10^5^ cells/ml in 10 ml DMEM supplemented as described above. Cells were treated with 0, 2, or 10 mM succinate for 5 min. Cell collection, protein estimation, separation, and transfer were done as previously reported (45). Antibodies were rabbit anti-phospoERK (#4370 Cell Signaling, Danvers, MA, USA), rabbit anti-ERK antibody (AF1576 R&D Systems, Minneapolis, USA), goat anti-GFP (AB0020 Sicgen-Research and Development in Biotechnologa Ltd, Carcavelos, Portugal), goat anti-mCherry (AB0040 Sicgen), or mouse anti-β-actin (A1978 Sigma-Aldrich). Appropriate peroxidase-labeled secondary antibodies (Dako) were used. Visualization was achieved by chemiluminescence detection using Amersham ECL Prime Western Blotting Detection Reagent (GE Healthcare, Freiburg, Germany) in a Fusion SL imaging system (Vilber Lourmat, Eberhardzell, Germany). Band intensity was determined by optical density analysis using image J (Rasband, W.S., ImageJ, U. S. National Institutes of Health, Bethesda, MD, USA, https://imagej.nih.gov/ij/, 1997-2016).

Proteins of hPheo1-Ctr, -*SDHB*^KO23^, and -*SDHB*^KO23Rec^ were harvested and blotted as previously described (46). The following primary antibodies were used: anti-GAPDH (Cell Signaling, #5174), anti-SDHA (Abcam, ab14715), anti-SDHB (Abcam, ab14714). HRP-conjugated secondary antibodies were used in TBS/tween with 5% non-fat dried milk for 1 h at room temperature. Protein bands were quantified using AzureSpot 2.0 software (Azure Biosystems).

### Confocal Microscopy

Cells were grown in Lab-Tek II chamber slides (Thermo Fisher Scientific, coated with rat tail collagen (Sigma-Aldrich, Taufkirchen, Germany), as previously described (47) and fixed in 4% paraformaldehyde (Electron Microscopy Sciences, Hatfield, PA, USA) after washing in PBS (Gibco). Cells were incubated in 300 nM DAPI solution to visualize cell nuclei (Invitrogen, Thermo Fischer Scientific). After washing cells were coverslipped in a solution containing 12% mowiol 4-88 (Calbiochem, EMD Chemicals, Inc., Gibbstown, NJ, USA), 30% glycerol, 2.5% 1,4- diazobicyclo-[2.2.2]-octane (DABCO) (Sigma- Aldrich), in 0.12 M tris, pH 6.8. A TCS SP5 confocal microscope (Leica, Wetzlar, Germany) with HCX PL APO CS 63× oil UV corrected objective, aperture 1.4, scanning frequency 100 Hz, average 4× and pinhole 1 AU was used to take representative images.

### Cell Viability

Stably transfected PC12 cells were seeded at 10,000 cells/well into collagen-1-coated 96-well plates in 100 µl supplemented DMEM media. The following day, cells were treated with sodium succinate (Sigma-Aldrich) at 0.5, 1, 2, 10, or 20 mM in supplemented media or supplemented media alone as control. Media pH was unaffected by succinate at the indicated concentrations. Cell viability was measured after 24 h and 48 h using an XTT-based cell proliferation kit (PromoKine, PromoCell, Heidelberg, Germany). Signal was detected with a microplate reader (Spectrostar Nano, BMG Labtech, Ortenberg, Germany) at 450 nm and 630 nm 4 h after addition of 25 µl reaction solution per well.

Candidate SUCNR1 inhibitors were kindly provided by Prof. Guang-Bo Ge (**Table 1**). Cells were treated with the inhibitors for 48 h in the presence or absence of 10 mM succinate, after which cell viability was determined.

**Table 1:** Structure and purity of SUCNR1 inhibitors.

No.	MW	Structure	Purity
**1**	**458.40**	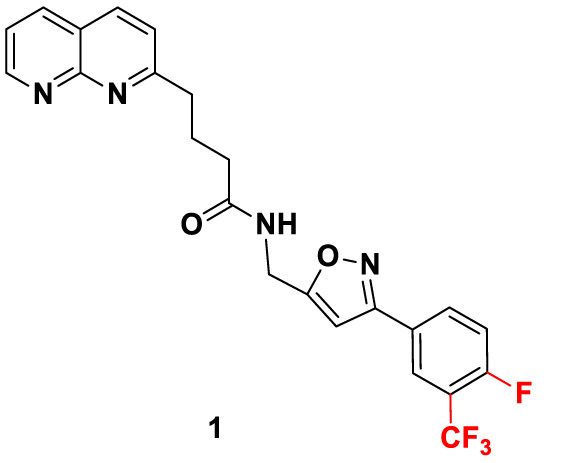	**98.3%**
**2**	**440.42**	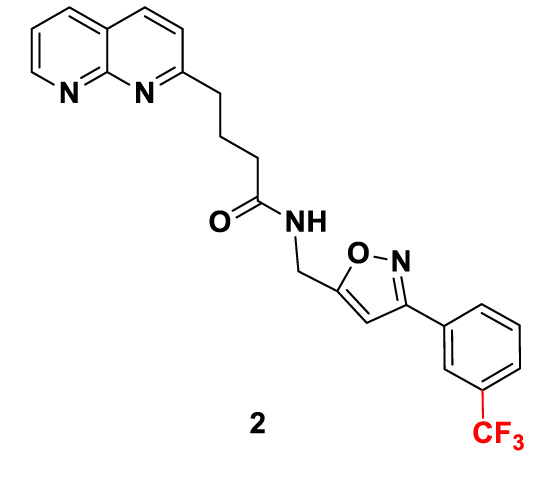	**98.4%**
**3**	**386.45**	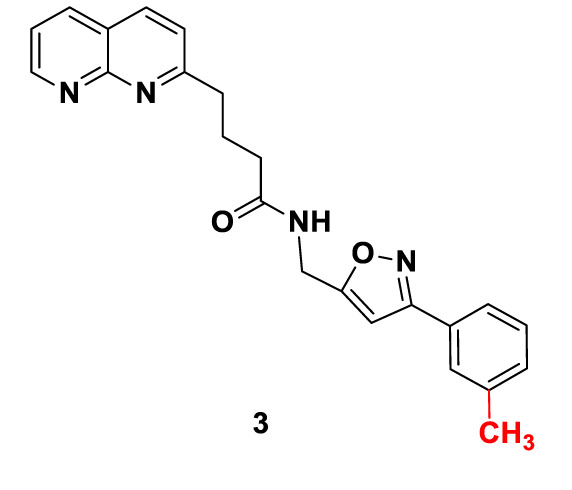	**98.1%**

### SUCNR1 Inhibitors

SUCNR1 inhibitors have been synthesized following previously published protocols (37). Compound 1 corresponds to compound 5 g from the cited reference. Structures and purities are listed in **Table 1**.

### Statistics

Statistic evaluation was performed using SPSS, Stata, or Prism. ANOVA, or multivariate ANOVA was performed with Dunnett’s or LDS post-hoc analysis, as indicated.

## Results

### SUCNR1 Expression in Human PPGLs

**Figure 1:**
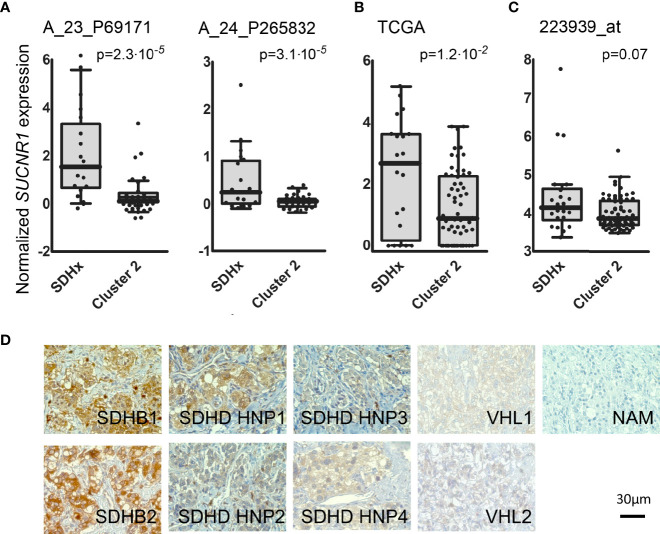
**(A)** Box and whisker Tukey plots showing the expression of *SUCNR1* in PPGLs of three published series. Data from GSE19422 and GSE51081 (38, 39), showing two different probes for *SUCNR1*. SDHx (n = 19: 10 *SDHB*, 3 *SDHC*, 6 *SDHD*), cluster 2 (n = 37: 3 *FGFR1*, 7 *HRAS*, 3 *MAX*, 5 *NF1*, 16 *RET*, and 3 *TMEM127*). **(B)** Data from the TCGA project (41), SDHx (n = 20: 17 *SDHB*, 3 *SDHD*) and cluster 2 (n = 61: 2 *FGFR1*, 17 *HRAS*, 2 *MAX*, 22 *NF1*, 17 *RET*, and 1 *TMEM127*); **(C)** Data from E-MTAB-733 (40), SDHx (n = 23: 1 *SDHA*, 16 *SDHB*, 1 *SDHB+SDHA*, 2 *SDHC*, 3 *SDHD*) and cluster 2 (n = 67: 2 *FGFR1*, 6 *HRAS*, 2 *MAX*, 36 *NF1*, 17 *RET*, 1 *TMEM127*, and three tumors with mutations in two different drivers: *MAX*+*HRAS*, *NF1*+*FGFR1*, and *RET*+*SDHA*). One-tailed Mann-Whitney test was applied to test for significant differences. **(D)** SUCNR1 protein expression determined by immunohistochemical staining in human PPGL tissue and normal adrenal medulla. Paraffin embedded PPGL samples from patients with mutations in succinate dehydrogenase B (*SDHB*, n = 2), succinate dehydrogenase D (*SDHD*, n = 4), the von-Hippel-Lindau gene (*VHL*, n = 2) as well as one sample of normal adrenal medulla (NAM) were used. *SDHD* PGLs were from the head and neck area (HNP).

In a previous microarray study, we detected elevated mRNA expression of *SUCNR1* in *SDHB* PPGLs and *SDHD* HNPs compared to normal adrenal medulla (36). Here we show that *SUCNR1* displays higher expression in *SDHx* PPGLs compared to cluster 2 tumors (**Figures 1A–C**). Cluster 2 PPGLs have a far lower risk of metastatic disease and are characterized by activation of kinase-signaling. Immunohistochemical staining of human PPGL tissues with different hereditary backgrounds confirmed elevated SUCNR1 protein expression in *SDHB* PPGLs and *SDHD* HNPs, compared to *VHL* pheochromocytomas. Normal adrenal medulla barely showed a *SUCNR1* signal (**Figure 1D**).

### SUCNR1 in Chromaffin Cells

*Sucnr1* expression was evaluated in established chromaffin cell models. However, qRT-PCR revealed very low mRNA levels in MPC, MTT, PC12, and hPheo1 (Ct >30 at 30–50 ng template load).

In HepG2 cells, *SDHB* silencing and succinate treatment have been shown to induce *SUCNR1* expression (35). We thus evaluated succinate levels and *Sucnr1* expression in previously prepared MTT cells silenced for *Sdhb* (43). The succinate to fumarate and succinate to citrate levels were increased by 1.6–2.4-fold in sh*Sdhb* cells compared to control cells, while the fumarate levels were mainly similar in all cell types (**Supplementary Figure S1A**). No significant difference was observed in *Sucnr1* expression level (**Supplementary Figure S1B**). To better model the situation observed in human PPGL tissue of 25-fold elevated succinate and 80% decreased fumarate (11), we exposed the cells to hypoxia for 24 h (1 and 10% O_2_), as has been previously effectively performed (48). As hypothesized (49), hypoxia augmented succinate accumulation and fumarate depletion particularly in the sh*Sdhb* cells, leading to an increase of the succinate to fumarate ratio (**Supplementary Figure S1A**). Nevertheless, the still mild succinate accumulation did not significantly induce *Sucnr1* mRNA expression (**Supplementary Figure S1B**).

**Figure 2:**
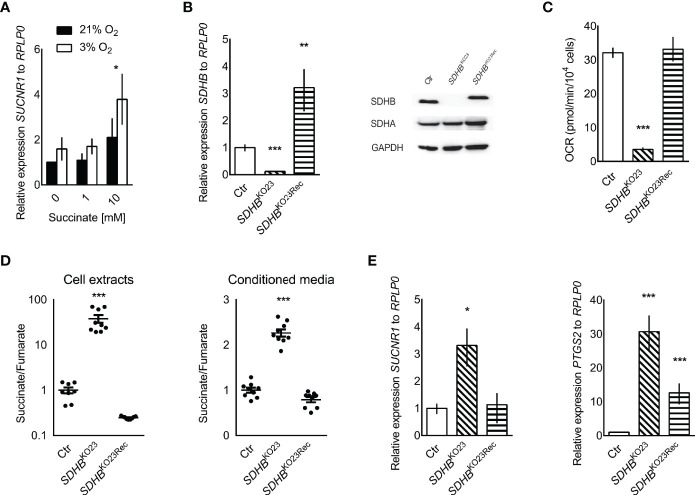
**(A)** Relative *SUCNR1* mRNA expression in hPheo1 treated with succinate or oxygen deprivation (n = 3). Three-way ANOVA showed significant differences for oxygen (p = 0.033) and treatment (p = 0.014), after verifying there was no interaction between oxygen and treatment. Dunnett’s *post-hoc* test was performed for the treatment main effect. The * above the 10 mM succinate bars reflects the significant difference from 0 mM, p = 0.022. **(B)** Relative expression of *SDHB* mRNA in hPheo1 parental cells (Ctr), *SDHB* knockout (*SDHB*^KO23^), and *SDHB* knockout cells re-expressing SDHB (*SDHB*^KO23Rec^). Representative Western blot for SDHB, SDHA, and GAPDH in hPheo1-Ctr, -*SDHB*^KO23^, -*SDHB*^KO23Rec^ (right). SDHB protein expression was diminished in hPheo1-*SDHB*^KO23^ and normalized in -*SDHB*^KO23Rec^ with re-constitution of human SDHB-FLAG. **(C)** Basal oxygen consumption rate of hPheo1-Ctr, -*SDHB*^KO23^, -*SDHB*^KO23Rec^ as determined by Seahorse XF analyzer. Basal respiration measured as oxygen consumption rate was significantly decreased in *SDHB*^KO23^ and normalized with *SDHB* re-constitution (n = 2). **(D)** Succinate-to-fumarate ratios in cell extracts (left) of hPheo1-Ctr, -*SDHB*^KO23^, -*SDHB*^KO23Rec^ and conditioned media (right) (n = 3, each). Data are shown as mean ± SEM. **(E)** Relative mRNA expression of *SUCNR1* and *PTSG2* in hPheo1-Ctr, -*SDHB*^KO23^, and -*SDHB*^KO23Rec^. ANOVA with Dunnet’s *post hoc* test for difference from Ctr was performed for delta Cts. *p ≤ 0.05, **p ≤ 0.01, ***p ≤ 0.001 (n = 3).

Interestingly, treatment of hPheo1 with external succinate at 10 mM or exposure to 3% oxygen for 24 h significantly increased *SUCNR1* expression (**Figure 2A**). A three-way ANOVA revealed no interaction for succinate and oxygen. Replicate and oxygen factors were coded as categorical, while the succinate level was coded with continuous values of 1/2/3, to reflect the expected ordered impact of increasing succinate dose, showing significant differences for oxygen (p = 0.033) and treatment (p = 0.014). Dunnett’s *post-hoc* test on treatment main effect showed that the 0 and 10 mM succinate levels differed with p = 0.022.

To evaluate causality of SDHB dysfunction, *SDHB* was knocked out in hPheo1. Successful knockout and re-expression are shown by qRT-PCR and Western blot (**Figure 2B**). Respiration was vastly decreased in hPheo1-*SDHB*^KO23^ compared to the parental and -*SDHB*^23Rec^ cells (**Figure 2C**). Succinate to fumarate levels from cell extracts showed a mean 40-fold increase of succinate to fumarate (**Figure 2D**). Excess succinate was released to the media, as evident by a doubling of the succinate to fumarate ratio.

*SDHB* deficient hPheo1 showed significantly increased *SUCNR1* expression (p = 0.018, **Figure 2E**). *PTGS2*/*COX2* a downstream effector of SUCNR1 signaling in inducible pluripotent neural stem cells (50) and retina in diabetic rats (29) was also significantly increased in hPheo1-*SDHB*^KO23^ and to a much smaller extent in -*SDHB*^KO23Rec^ (**Figure 2E**).

### Succinate Promotes Proliferation *via* SUCNR1

**Figure 3:**
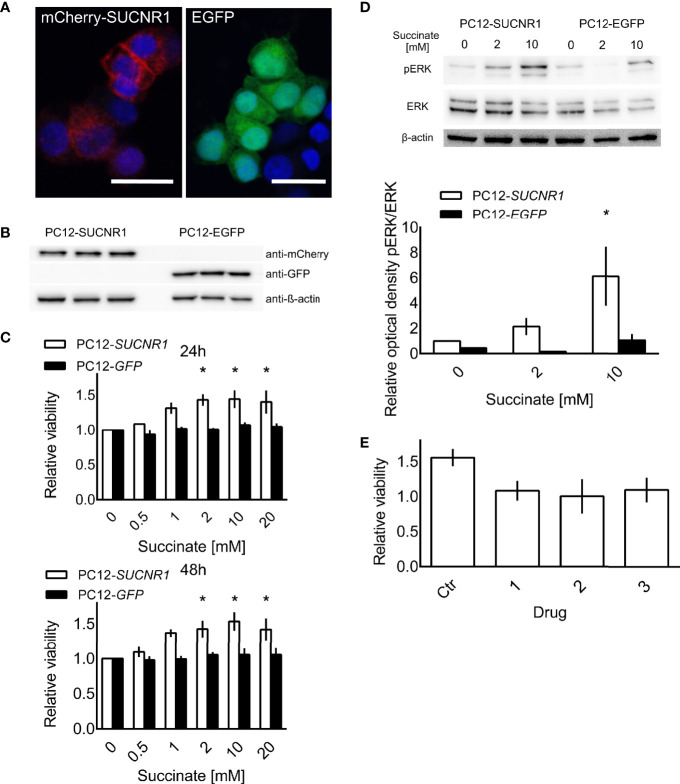
PC12 cells transfected with a fusion protein of *mCherry* and *SUCNR1* or *EGFP*. **(A)** Confocal microscopy confirmed punctate mCherry signal in accordance with cell surface location typical for G-protein coupled receptors, while EGFP was equally distributed throughout the cells. **(B)** Western blot for mCherry and GFP confirmed successful transfection. **(C)** Cell viability of transfected cells was determined by XTT assay after 24 and 48 h of exposure to the indicated succinate concentrations. ANOVA with *post-hoc* Dunnet’s test for difference from 0 mM succinate treatment was performed. *p < 0.05 (n = 4). **(D)** Representative Western blot showing increased ERK phosphorylation in *SUCNR1* transfected cells after 5 min exposure to 2 mM or 10 mM succinate, while no difference in phospho-ERK could be determined in *EGFP* transfected cells (n = 3). Mean optical density ratios of phospho-ERK to ERK ± SEM of three independent experiments are shown as bar graph. Three-way ANOVA of the log transformed pERK/ERK ratios revealed significant interaction between cell type and succinate concentration (p = 0.045). ANOVA for the effect of treatment in PC12-SUCNR1 was significant at p = 0.006, with Dunnet’s *post-hoc* test indicating a significant difference in phosphorylation at 10 mM succinate compared to control (p = 0.023). In PC12-EGFP cells, succinate had no effect on ERK phosphorylation (p = 0.454). **(E)** PC12-SUCNR1 cells were treated with candidate inhibitors **(A**–**C)** in presence and absence of succinate. The bars show the relative viability of cells treated with the drug or vehicle and succinate relative to drug or vehicle alone (n = 2). Data are shown as mean ± SEM.

To explore SUCNR1 related effects in PPGL cells independent of intracellular succinate accumulation, we stably transfected PC12 cells with human *SUCNR1*. Confocal microscopy revealed a punctate staining pattern, which is in line with cell surface expression of the mCherry-hSUCNR1 fusion protein, while EGFP was equally distributed in control transfected cells, indicating cytosolic localization (**Figure 3A**). Western blot for mCherry and EGFP showed strong bands in the transfected cells, with no signal in the respective counterparts (**Figure 3B**).

Treatment of *SUCNR1*-transfected PC12 with 2, 10, or 20 mM succinate significantly increased cell viability compared to untreated controls after 24 and 48 h of treatment. Cell viability of *EGFP*-transfected PC12 did not change in response to succinate treatment (**Figure 3C**). Furthermore, SUCNR1-stimulation with 10 mM succinate significantly induced ERK-phosphorylation in *SUCNR1-*, but not *EGFP*-transfected cells (**Figure 3D**). Simultaneous treatment of *SUCNR1*-PC12 cells with 10 mM succinate and 10 nM of one of three candidate succinate receptor inhibitors successfully reversed the increase in relative viability of *SUCNR1*-PC12 treated with 10 mM succinate alone (**Figure 3E**).

## Discussion

*SUCNR1* expression is induced by hypoxia, extracellular succinate, and loss of *SDHB* in hPheo1, and SUCNR1 signaling increases viability in PC12 cells. Taken together, these data suggest that accumulating succinate in *SDHx* PPGLs may have a previously unrecognized oncometabolic effect by stimulating SUCNR1 in an autocrine manner.

In several cell types and tissues, SUCNR1 expression has been induced or correlated with *SDHB* silencing, succinate treatment, or hypoxia (35, 51, 52). However, differences in susceptibility or interfering mechanisms may exist. In MTT sh*Sdhb* cells succinate only slightly accumulated. However, under hypoxia, an up to 30-fold increase in succinate to fumarate ratio was reached in sh*Sdhb*64. Nevertheless, expression of *Sucnr1* was not significantly induced. At an only slightly higher 40-fold increase seen in hPheo1 *SDHB*^KO23^, *SUCNR1* was significantly up-regulated. Interestingly, in hPheo1 *SUCNR1* induction was also achieved by treatment with 10 mM extracellular succinate or 3% oxygen. If the discrepancy we observed between MTT and hPheo1 is due to cell specific reasons or the amount of succinate accumulation remains unclear. Other cell models with similarly or even more efficient succinate accumulation have been reported (48, 53, 54), however SUCNR1 expression has not been evaluated. Highly likely, extracellular succinate stimulation of the receptor leads to positive feedback on its expression, which can only be reached by substantial increase in extracellular succinate due to severe SDH inhibition or hypoxia. Here we show that hPheo1 *SDHB*^KO23^ release excess succinate into the media, which is probably related to the amount of succinate accumulation. Surprisingly, *SUCNR1* was not elevated in *SDHD* abdominal and thoracic PGLs in our microarray study, while expression was increased in *SDHD* HNPs and *SDHB* PPGLs (36). Succinate to fumarate levels have been shown to be lower in *SDH*x HNPs compared to adrenal or extra-adrenal localization (11). Thus, additional factors likely influence *SUCNR1* expression in PPGL tissue. Potentially, tumor tissue pH and monocarboxylate transporter 1 expression level play an essential role, as these highly likely determine succinate release to the extracellular space (20, 21). Of note, hypoxia or HIF activation positively regulate monocarboxylate transporter 1 expression [summarized in (55)].

It will be of major interest for future studies to evaluate discrepancies between the models in more detail, also with respect to dysfunction of other SDH subunits. However, to date no comparable models with knockout of the different subunits is available (56).

Analysis of publically available data from three large mRNA expression studies showed a significant increase or strong trend towards increased *SUCNR1* expression in *SDHx* compared to cluster 2 PPGLs (**Figures 1A–C**). Differences in composition of the *SDHx* cohorts with respect to exact mutation, level of succinate accumulation, and tumor location likely contribute to the variance between the cohorts.

While the stimulatory concentration of succinate in the millimolar range may appear high, such high levels can be expected in *SDHx* PPGLs (11). The median concentration of succinate in human *SDHx*-deficient PPGLs was close to 1 µg/mg tissue. With the molecular weight of succinate of 118.09 g/mol and an estimated density of PPGL tissue at the same level as normal adrenal [1.03 g/ml (57)], the tissue succinate content can be estimated at 8.7 mM. This is in the same range as the pro-proliferative dose of 2–20 mM used in our experiments.

Previously, ERK1/2 activation as well as induction of *PTGS2* expression and/or prostaglandin E2 release have been reported as downstream effectors of SUCNR1 signaling (25, 29, 31, 50). Expression of PTGS2/COX2 has been evaluated in PPGLs, however no clear relation with genetic background was evident (58). As a hypoxia responsive gene, induction of *PTGS2* in hPheo1 *SDHB*^KO23^ may not entirely depend on SUCNR1 activation, yet may be worthwhile to further explore. Further roles of SUCNR1 on metastatic spread, immune-modulation and chemotaxis, or tumor angiogenesis, as observed in other tissues (30, 31, 59), remain to be evaluated in *SDHx* PPGLs.

Our data indicate that SUCNR1 mediated proliferation enhancement can be disrupted by targeted treatment with SUCNR1 inhibitors. Three compounds generated to inhibit SUCNR1 (Drugs 1, 2, 3) were available to us. Drug 1 corresponds to the previously described small molecule inhibitor 5 g, which shows excellent receptor binding capabilities and selectivity (37). Drugs 2 and 3 are new derivatives of Drug 1. Pharmacokinetic parameters of compounds closely related to drug 1, such as oral bioavailability and clearance (0.12–0.17 nmol/min/kg) are favorable. Plasma concentrations of 37–70 µM have been reached. Selectivity was at least 100-fold increased over binding to the closely related GPR99 (37). It has been argued that newly developed SUCNR1 agonists may be superior to investigate the role of SUCNR1 as these agonists activate the SUCNR1 without the additional metabolic functions of succinate (60, 61). Regardless, the confounding effect of succinate on cell viability in PC12 cells should be negligible, since *EGFP*-transfected control cells were not influenced by succinate treatment. Expression of SUCNR1 was considerably higher in *SDHB* PPGL and *SDHD* HNP tissue than normal adrenal medulla. Thus, normal adrenal medulla will most likely not be affected by treatment with SUCNR1 inhibitors. However, vulnerability of normal adipocytes, hepatocytes, retinoblasts, or other SUCNR1 expressing cells to systemic application of SUCNR1-inhibitors remains to be evaluated together with potential immunomodulatory effects.

SUCNR1 inhibition may provide a promising new treatment approach for the aggressive and often inoperable *SDHx* tumors. Effectiveness of these novel drugs may likely be extended to unresectable or metastatic *SDH*-deficient renal cell carcinomas, gastrointestinal stroma tumors, thyroid, and pancreatic neuroendocrine tumors, or other conditions exhibiting disturbed SUCNR1-signaling due to hypoxia or hyperglycemia.

## Data Availability Statement

The raw data supporting the conclusions of this article will be made available by the authors, without undue reservation.

## Ethics Statement

The studies involving human participants were reviewed and approved by the *Eunice Kennedy Shriver* National Institute of Child Health and Human Development’s Institutional Review Board. The patients/participants provided their written informed consent to participate in this study.

## Author Contributions

Conceptualization, SF, KP, ZZ, and HL. Methodology and validation, DM, KHV, NB, SR, LA, RA, JF, MM, G-BG, and SF. Formal analysis, DM, JF, AM-M, BC, RW, and SF. Investigation, DM, KHV, NB, SR, LA, and SF. Resources, NB, SR, KHV, JN, MD, G-BG, PD, MM, RA, KP, and HL. Data curation, DM, NB, SR, AM-M, BC, JF, KHV, and SF. Writing—original draft preparation, DM and SF. Writing—review and editing, DM, NB, KHV, SR, SF, PD, HL, and KP. Visualization, DM, KHV, JF, and SF. Supervision, KP, HL, and SF. Funding acquisition, SF, KP, and HL. All authors contributed to the article and approved the submitted version.

## Funding

This study was supported in part by the Intramural Research Program of the *Eunice Kennedy Shriver* NICHD, NIH, MD, and the University Medical Center Schleswig-Holstein, Lübeck, Germany. DM received a stipend for excellence in medicine from the University of Lübeck. NB, SR, MD, and PD were supported by a grant from the Paradifference foundation.

## Conflict of Interest

The authors declare that the research was conducted in the absence of any commercial or financial relationships that could be construed as a potential conflict of interest.

## Supplementary Material

Figure S1 **(A)** Metabolite levels in MTT shSdhb and control cells under normoxia and at 1% and 10% oxygen. The normoxia control cells that were kept in parallel to the 1% oxygen condition are labelled N1, the control cells from the 10% oxygen condition are labelled N10. The succinate to citrate, fumarate to citrate, and succinate to fumarate levels are shown from top to bottom. The bars show means ± SEM of n=4 (1% oxygen) and n=5 (10% oxygen) independent experiments. 2-way ANOVA showed significant differences between cell types and oxygen conditions. P-values for LDS post-hoc statistics of ANOVA for main effects are shown. Lower case letters indicate significant differences between oxygen concentrations for each cell type. Replication of x indicate 1: p≤0.05, 2: p≤0.01, 3: p≤0.001. Asterisks indicate significant difference between cell types within a given oxygen condition. * indicates p≤0.05, ** indicates p≤0.01, *** indicates p≤0.001. **(B)** Relative expression of Sucnr1 to Rplp0 in cells kept at 10% (top) and 1% (bottom) oxygen with respective normoxia controls. There was no statistic difference (n=3).
