# Supplementary figures and images for: Succinate Mediates Tumorigenic Effects via Succinate Receptor 1: Potential for New Targeted Treatment Strategies in Succinate Dehydrogenase Deficient Paragangliomas
Source: Front Endocrinol (Lausanne). 2021 Mar 12;12:589451. doi: 10.3389/fendo.2021.589451 (PMC7994772; doi:10.3389/fendo.2021.589451)

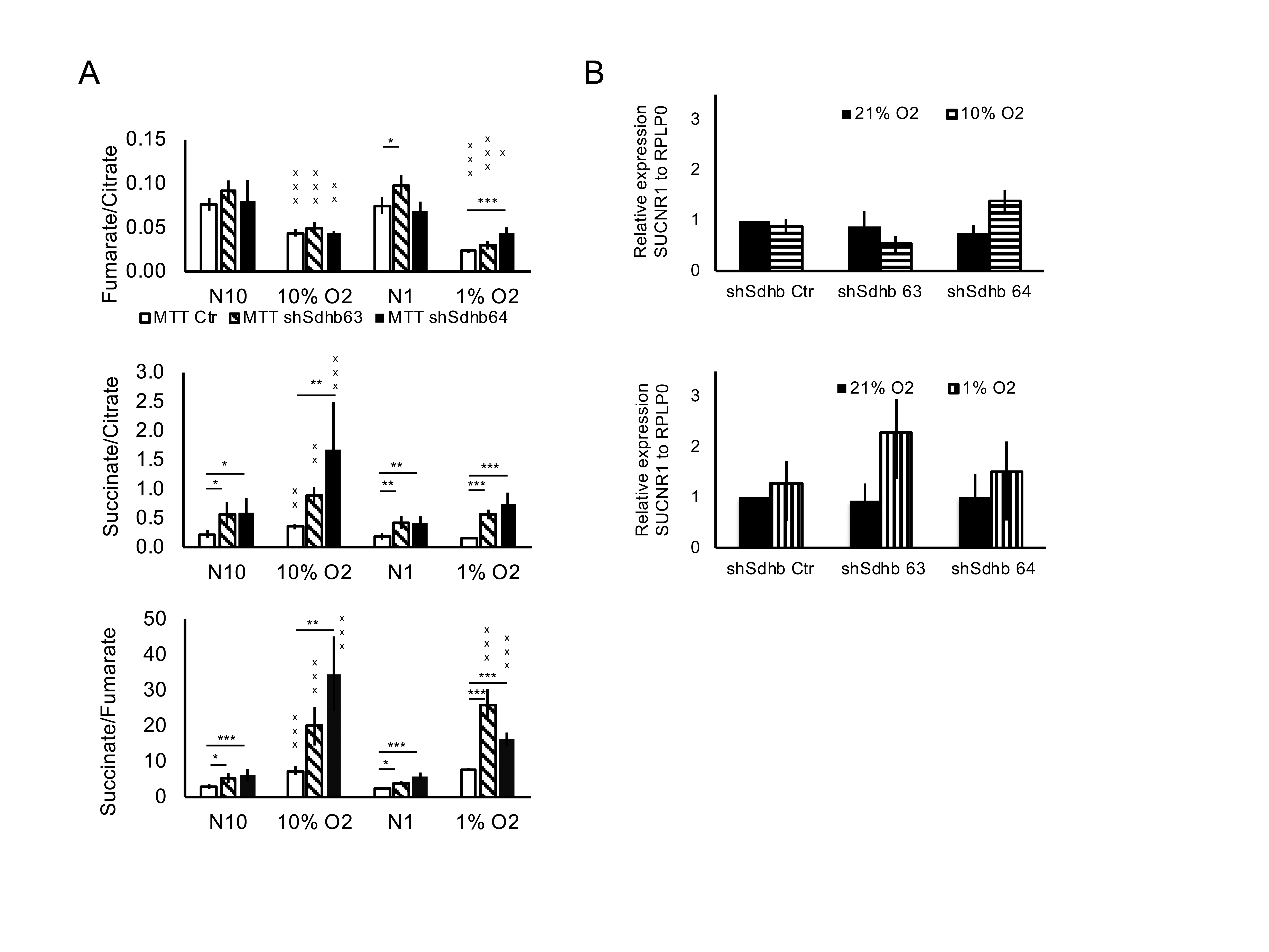

Supplement: Figure S1 — (A) Metabolite levels in MTT shSdhb and control cells under normoxia and at 1% and 10% oxygen. The normoxia control cells that were kept in parallel to the 1% oxygen condition are labelled N1, the control cells from the 10% oxygen condition are labelled N10. The succinate to citrate, fumarate to citrate, and succinate to fumarate levels are shown from top to bottom. The bars show means ± SEM of n=4 (1% oxygen) and n=5 (10% oxygen) independent experiments. 2-way ANOVA showed significant differences between cell types and oxygen conditions. P-values for LDS post-hoc statistics of ANOVA for main effects are shown. Lower case letters indicate significant differences between oxygen concentrations for each cell type. Replication of x indicate 1: p≤0.05, 2: p≤0.01, 3: p≤0.001. Asterisks indicate significant difference between cell types within a given oxygen condition. * indicates p≤0.05, ** indicates p≤0.01, *** indicates p≤0.001. (B) Relative expression of Sucnr1 to Rplp0 in cells kept at 10% (top) and 1% (bottom) oxygen with respective normoxia controls. There was no statistic difference (n=3). [file Image_1.tif]
