# Supplementary material for: Succinate Mediates Tumorigenic Effects via Succinate Receptor 1: Potential for New Targeted Treatment Strategies in Succinate Dehydrogenase Deficient Paragangliomas
Source: Front Endocrinol (Lausanne). 2021 Mar 12;12:589451. doi: 10.3389/fendo.2021.589451 (PMC7994772; doi:10.3389/fendo.2021.589451)
Supplement: Supplementary file 2 [file Table_1.docx]

**Table S1.** Parallel reaction monitoring transitions and scan parameters for targeted analysis of succinate and fumarate in hPheo1-Ctr, -*SDHB*^KO23^ and -*SDHB*^KO23Rec^

|  | Precursor [M-H]^-^ | Fragment (Quantifier) | Normalized collision energy [eV] | Spray voltage [kV] | Sheath/auxiliary gas [arb unit] | Auxiliary gas temperature [°C] |
| --- | --- | --- | --- | --- | --- | --- |
| Succinate | 117.019 | 71.014 | 30 | 3 | 30/10 | 80 |
| Fumarate | 115.004 | 73.030 | 30 | 3 | 30/10 | 80 |

**Table S2.** Sybr green primer pairs.

| **Species** | **Target** | **forward** | **reverse** |
| --- | --- | --- | --- |
| Human | *RPLP0* | CCAGCTCTGGAGAAACTG | CTTCACATGGGGCAATGG |
| Human | *SUCNR1* | TgTgTcTAAcAcTgTTggggTTcc | TccTcAcATTccgcATgAcg |
| Human | *PTGS2* | CAAATTGCTGGCAGGGTTGC | AGGGCTTCAGCATAAAGCGT |
| Human | *SDHB* | GAAACTGGACGGGCTCTACG | GTCTCCGTTCCACCAGTAGC |
| Rat | *Rplp0* | TTGAAATCCTGAGCGATGTGCAGC | GCCATTGTCAAACACCTGCTGGAT |
| Rat | *Sucnr1* | ACAGCTGTCGCCCTTTTCTA | TCATGCCAACCTCTACACCA |
| Mouse | *Rplp0* | GAGGACCTCACTGAGATTCG | CTGGAAGAAGGAGGTCTTCTC |
| Mouse | *Sucnr1* | TgTgAgAATTggTTggcAAcAg | TcggTccATgcTAATgAcAgTg |
